# Opium and Cigarette Smoking are Independently Associated with Bladder Cancer: The Findings of a Matched Case - Control Study

**DOI:** 10.31557/APJCP.2021.22.10.3385

**Published:** 2021-10

**Authors:** Zahra Abdolahinia, Hamid Pakmanesh, Moghaddameh Mirzaee, Azam Bazrafshan, Mehdi Shafiei Bafti, Armita Shahesmaeili

**Affiliations:** 1 *HIV/STI Surveillance Research Center, and WHO Collaborating center for HIV surveillance, Institute for Futures Studies in Health, Kerman University of Medical Sciences, Kerman, Iran. *; 2 *Department of Urology, Faculty of Medicine, Kerman University of Medical Sciences, Kerman, Iran. *; 3 *Modeling in Health Research Center, Institute for Futures Studies in Health, Kerman University of Medical Sciences, Kerman, Iran. *; 4 *Kerman Population-based Cancer Registry (KPBCR), Deputy of Health, Kerman University of Medical Sciences, Kerman, Iran. *; 5 *Department of Communicable Diseases, Kerman University of Medical Sciences, Kerman, Iran. *

**Keywords:** Opioid, Cigarette smoking, bladder neoplasm, Iran, Kerman

## Abstract

**Background:**

Bladder cancer disproportionally affects the communities. While it is the ninth most common cancer in the world, in some parts of Iran including Kerman province it is the most common cancer among men. This study aimed to determine potential risk factors of bladder cancer in Kerman province, Iran.

**Methods:**

During February to July 2020, in this matched hospital-based case-control study, 100 patients with bladder cancer and 200 healthy individuals (matched in age and sex) were recruited. Socio-demographics status, occupational exposures, common diet, history of drug use and family history of cancer, were collected using a structured questionnaire. Bivariable and multivariable logistic regression were applied and crude and adjusted odds ratios (AOR) along with their 95% confidence intervals (95%CI) were calculated. Data were analyzed using Stata version 14 software.

**Results:**

Opium consumption, cigarette smoking and low level of income were associated with increased chance of bladder cancer. Compared to never use, use of opium up to 18000 Gram -year was associated with increased chance of bladder cancer (AOR: 6; 95% CI =2.3, 15.5). The chance was higher among those who used opium more than 18,000 Gram - year (AOR: 11.3; 95% CI =2.3, 15.5). In comparison with never smokers, the chance of bladder cancer increased among those who smoked up to 20 pack-year cigarette) (AOR: 3.4; 95%CI= 1.3, 8.9) and those who smoke ≥ 20 pack-year (AOR: 15.8; 95% CI= 5.9, 42.4).Conclusions: The observed strong dose-response association between opium consumption, cigarette smoking and bladder cancer highlights the need for extension of harm reduction programs especially in regions with high burden of disease.

## Introduction

Bladder cancer is the ninth most common cancer globally, affecting more than 430,000 men and imposing a high social and economic burden on individuals and health care systems globally (Khazaei et al., 2019), especially among men and the elderly (Miller et al., 2018). In 2018, it was estimated that approximately 3% of all newly diagnosed cancers and 2.1% of all cancer-related deaths globally were due to bladder cancer (Khazaei et al., 2019). 

In Iran, bladder cancer is showing an increasing trend (Hassanipour et al., 2019), with more than 70,000 new cases and 30,000 registered deaths annually (Ahmadi et al., 2012). It is the fifth most common cancer among Iranian men (Farmanfarma et al., 2020). However, there are some variations in the incidence of bladder cancer between geographical areas. Kerman, a province located in southeast Iran, with an age-standardized incidence rate (ASR) of 24.6 per 100,000 population – compared to the ASR of 10.9 for the whole country (Hassanipour et al., 2019) – is one of the areas where bladder cancer is most prevalent (Shahesmaeili et al., 2018). Disparities in environmental, contextual, and individual factors may explain the high incidence of bladder cancer in Kerman. However, the studies on assessing underlying factors are scarce. 

A variety of factors, including behavioral, environmental, and occupational risk factors, have been associated with the increased risk of this cancer. Tobacco use, opium consumption, some occupational exposures such as exposure to aromatic amines and black carbon, which are produced in painting, the plastics industry, and coal mines, positive family history, diet, and prolonged consumption of water contaminated with arsenic or chlorine are among the main risk factors (Koutros et al., 2016; Bravi et al., 2018; Wong et al., 2018). 

Kerman province is one of the provinces richest in mineral resources in the country, and the city of Kerman is surrounded by a variety of active mines, including copper, coal, iron, and titanium mines (Najafipour et al., 2015). Furthermore, the latest studies in Kerman province indicate that the mean concentration of groundwater heavy metals such as arsenic in some districts is higher than the standard value (Malakootian and Mohammadi Senjedkooh, 2014; Nazari and Abbasnejad, 2015). Additionally, the prevalence of opium consumption, a known risk factor of bladder cancer, in Kerman province is higher than the national average (21%) (Shahesmaeili et al., 2018).

Therefore, there is a wide range of possible risk factors in Kerman, explaining the higher rate of bladder cancer in this province. However, there is no comprehensive study in which the role of all possible risk factors and the potential interaction between them are studied simultaneously. As determining the principal risk factors and high-risk groups may be beneficial to the prevention, screening and early diagnosis of this disease, this matched case-control study aimed to identify the risk factors of bladder cancer in Kerman province.

## Materials and Methods

*Study design and participants*

In this matched hospital-based case-control study, 100 cases and 200 controls who were aged above 40 and had consented to participate in the study were recruited from February to July 2020. Cases were patients with confirmed bladder cancer who referred to the urology department of Bahonar Hospital, a teaching hospital affiliated with Kerman University of Medical Sciences, for treatment workup. For each case, we selected two controls: one from patients referred to the ophthalmology department of Shafa general hospital, Kerman, Iran, and another from patients referred to the urology department of Bahonar Hospital, Kerman, Iran, who had undergone cystoscopy due to diseases unrelated to the bladder; the absence of bladder tumors in controls selected from the urology department was confirmed by cystoscopy. The controls had no history of cancer, and cases and controls were matched based on age (± 5 years) and sex.

*Study instrument:*

Data were collected through face-to-face interviews using a structured questionnaire. The questionnaire consisted of 50 questions categorized into five sections: socio-demographics, occupational exposure, common diet, history of drug use (cigarettes, opium, hookah, and alcohol), and family history of cancer.

*Independent variables*

Pack years smoked was calculated using the following formula: pack-year = (number of cigarettes smoked per day ÷ 20 cigarettes) x number of years. Three groups were defined according to the pack-year: never smokers, light smokers (up to 20 pack-years), and heavy smokers (≥ 20 pack-years). Similarly, the amount of opium consumption was calculated based on gram-years and categorized into two groups: < 18,000 gram-years and ≥ 18,000 gram-years in lifetime. Other potential risk factors measured were: monthly income (< 20 vs. ≥ 20 million rials), education (< 12 vs. ≥ 12 years), use of alcohol (never vs. ever used), exposure to second-hand smoke (yes vs. no), age of smoking onset (< 20 vs. ≥ 20 years), age of first opium use (< 25 vs. ≥ 25 years), second hand exposure to opium (yes vs. no), use of hookah (yes vs. no), use of opium derivatives (yes vs. no), history of work in industrial factories (yes vs. no), history of work as a driver (yes vs. no), history of work as a farmer (yes vs. no), history of work as a construction worker (yes vs. no), residing in proximity to industrial zone (< 2 km vs. ≥ 2 km), exposure to chemical fertilizers (yes vs. no), use of grilled foods (< 1 time vs. 1–3 times vs. ≥ 4 times per week), use of stuck-pot (< 1 time vs. 1–3 times vs. ≥ 4 times per week), use of carbonated drinks and juices with preservatives (< 1 time vs. 1–3 times vs. ≥ 4 times per week), use of eggs (< 1 time vs. 1–3 times vs. ≥ 4 times per week), cereal (1–3 times vs. ≥ 4 times per week), and pickles (< 1 time vs. 1–3 times vs. ≥ 4 times per week).

*Statistical analysis*

Data were analyzed using Stata version 14. To explore factors associated with bladder cancer, we applied bivariate and multivariable logistic regression, and crude and adjusted odds ratios (AORs) along with their 95% confidence intervals (95% CI) were reported. Variables with a P-value < 0.2 in the bivariate analysis were entered into the multivariable regression model. The final model was reduced based on the F-test, and P-values < 0.05 were considered statistically significant. 

*Ethics*

The study protocol was reviewed and approved by the Research Review Board of the Kerman University of Medical Sciences (ethics code: IR.KMU.REC.1398.603). The risks and benefits of the study were explained to all eligible participants to obtain verbal informed consent; each potential participant had the choice to accept or refuse to participate in the study. All interviews were conducted anonymously in a private setting. 

## Results

*Characteristics of study participants*

In the present study, 300 participants (100 cases and 200 hospital controls) were recruited. Most participants were male (85.9% in cases, 83% in controls), educated less than 12 years (82.8% in cases, 70% in controls), earned less than 20 million rials (80.8% in cases, 62.5% in controls), and were married (89.9% in cases, 88% in controls). The mean ages of the cases and controls were 63.6 ± 9.65 and 59.3 ± 11 years, respectively (Table 1).

Overall, 71.7% of cases and 17% of controls had a history of cigarette smoking, and around 74.8% of cases and 14.5% of controls had used opium. In the majority of participants, the age at onset of cigarette smoking was ≥ 20 years (77.5% in cases vs. 79.4% in controls), and most participants had started opium use ≥ 25 years (73% in cases vs. 75% in controls). The distribution of other drugs used is summarized in Table 2.

*Bladder-cancer-associated factors*

Compared to controls, cases were less likely to be educated ≥ 12 years (%17.2 vs. 30%, P = 0.01), and earned ≥ 20 million rials (19.2% vs. 37.5%, P = 0.001). They were more likely to smoke ≥ 20 pack-years Cigarette in their life time (52.5% vs. 5%, P < 0.001), use ≥ 18,000 gram years opium in their lifetime (50.6% vs. 7.5%, P < 0.001), use alcohol (8.2% vs. 2%, P = 0.02), be exposed to second-hand smoke (40.4% vs. 27%, P = 0.02), have a history of working in a factory (10.1% vs. 4%, P = 0.03), reside near an industrial zone (18.2% vs. 9%, P = 0.03), be exposed to chemical fertilizers (41.4% vs. 27.5%, P = 0.01 ), have a history of working as driver (23.2% vs. 7%, P < 0.001), have a history of working as farmer (56.6% vs. 43.5%, P = 0.03), have a history of working as a construction worker (22.2% vs. 8%, P = 0.001), use grilled food 4–6 times per week (6.6% vs. 5.1%, P = 0.02), use carbonated drinks and juices with preservatives 4–6 times per week (47.5% vs. 33.3%, P = 0.03), use eggs < 1 times per week (16.1 vs. 6.0, P = 0.01), and use stuck-pot < 1 times per week (61.6% vs. 43.9%, P = 0.01) (Tables 2, 3, 4).

In the multivariable model, earning higher income was associated with a decreased likelihood of bladder cancer (AOR = 0.41 95%, CI = 0.18, 0.92), while opium consumption and cigarette smoking were associated with an increased chance of bladder cancer. Compared to those who had never used opium, the use of opium up to 18,000 gram-years in their lifetime was associated with an increased chance of bladder cancer (AOR = 6; 95% CI = 2.3, 15.5). The chance was even higher among those who used opium more than 18000 gram-years in their lifetime (AOR = 11.3; 95% CI = 2.3, 15.5). In comparison with those who had never smoked, the chances of developing bladder cancer increased among those who had smoked up to 20 pack-years of cigarette) (AOR = 3.4; 95%, CI = 1.3, 8.9) and those who smoked ≥ 20 pack-years (AOR = 15.8; 95% CI = 5.9, 42.4) (Table 4).

**Table 1:** Socio-Demographic Characteristics of 100 Cases with Bladder Cancer and 200 Hospital-Based Controls, in Kerman Province in 2020

variables	Case (n=100) N (%)	Control (n=200) N (%)	p-value
Sex			0.52
Female	14 (14.4)	34 (17)	
Male	85 (85.9)	166 (83)	
Age at interview			0.11
40-65	56 (56.6)	132 (66)	
≥65	43 (43.4)	68 (34)	
Education (years)			0.01
<12	82 (82.8)	140 (70)	
≥12	17 (17.2)	60 (30)	
Income			0.001
< 20 million Rials	80 (80.8)	125 (62.5)	
≥20 million Rials	19 (19.2)	75 (37.5)	
Marital status			0.70
Single	10 (10.1)	24 (12)	
Married	89 (89.9)	176 (88)	

**Table 2:** Cigarette Smoking, Opium and Alcohol Status of 100 Cases with Bladder Cancer and 200 Hospital-Based Controls, in Kerman Province in 2020

Variables	Case (n=100) N (%)	Control (n=200) N (%)	p-value
Cigarette smoking status			p <0.001
Never	28 (28.3)	166 (83)	
light smoker	19 (19.2)	24 (12)	
heavy smoker	52 (52.5)	10 (5)	
Opium consumption			p <0.001
Never	25 (25.2)	171 (85.5)	
< 18000 Gram- year	19 (19.2)	14 (7)	
≥18000 Gram- year	55(55.6)	15(7.5)	
Alcohol use			0.02
Never	90 (91.8)	196 (98)	
Ever used	8 (8.2)	4 (2)	
Age of first cigarette smoke			0.82
<20	16 (22.5)	7 (20.6)	
≥20	55 (77.5)	27 (79.4)	
Age of first opium use			0.83
<25	20 (27)	7 (25)	
≥25	54 (73)	21 (75)	
second hand exposure to smoking			0.02
No	59 (59.6)	146 (73)	
Yes	40 (40.4)	54 (27)	
Second hand exposure to opium			0.24
No	71 (72.5)	156 (78.8)	
Yes	27 (27.6)	42 (21.2)	
Ever used of hookah			0.83
No	96 (97)	193 (96.5)	
Yes	3 (3)	7 (3.5)	
Ever use of opium derivatives			0.33
No	96 (97)	198 (99)	
Yes	3 (3)	2 (1)	

**Table 3:** Occupational, environmental and Diet Status of 100 Cases with Bladder Cancer and 200 Hospital-Based Controls, in Kerman Province in 2020

Variables	Case (n=100) N (%)	Control (n=200) N (%)	p-value
History of work in industrial factories			0.03
No	86 (89.9)	192 (96)	
Yes	10 (10.1)	8 (4)	
Residing in proximity to industrial zone			0.03
< 2 km	81 (81.8)	182 (91)	
≥ 2 km	18 (18.2)	18 (9)	
Exposure to chemical fertilizers			0.01
No	58 (58.6)	145 (72.5)	
Yes	41 (41.4)	55 (27.5)	
Ever working as a driver			p <0.001
No	76 (76.8)	186 (93)	
Yes	23 (23.2)	14 (7)	
Ever working as a farmer			0.03
No	43 (43.4)	113 (56.5)	
Yes	56 (56.6)	87 (43.5)	
Ever working as construction worker			0.001
No	77 (77.8)	184 (92)	
Yes	22 (22.2)	16 (8)	
Use of grilled foods			0.02
< 1 time per week	58 (58.6)	86 (43.4)	
1-3 times per week	35 (35.4)	102 (51.5)	
≥4 times per week	6 (6. 6)	10 (5.1)	
Stuck-pot			0.01
< 1 time per week	61 (61.6)	87 (43.9)	
1-3 times per week	32 (32.3)	93 (47)	
≥4 times per week	6 (6.1)	18 (9.1)	
carbonated drink and juices with preservatives			0.03
< 1 time per week	20 (20.2)	63 (31.8)	
1-3 times per week	32 (32.3)	69 (34.9)	
≥4 times per week	47 (47.5)	66 (33.3)	
Use of egg			0.01
< 1 time per week	16 (16.1)	12 (6)	
1-3 times per week	60 (60.6)	135 (67.8)	
≥4 times per week	23 (23.2)	52 (26.1)	
Cereals			0.13
1-3 times per week	7 (7.1)	6 (3)	
≥4 times per week	92 (92.9)	193 (97)	
Pickle			0.08
< 1 time per week	64 (64.7)	103 (52)	
1-3 times per week	28 (28.3)	69 (34.9)	
≥4 times per week	7 (7.1)	26 (13.1)	

**Table 4:** Distribution of 100 Cases with Bladder Cancer and 200 Hospital-Based Controls, ORs, and Corresponding 95 % CIs by Association Factors in Kerman Province in 2020

Variables	Case (n=100) N (%)	Control (n=200) N (%)	Crude OR (95% CI)	p-value	Adjusted OR (95% CI)	p-value
Income				0.001		0.032
< 20 million Rials	80 (80.8)	125 (62.5)	Referent		Referent	
≥20 million Rials	19 (19.2)	75 (37.5)	0.39 (0.22-0.70)		0.41 (0.18-0.92)	
Education (year)				0.01	_*	_
<12	82 (82.8 )	140 (70)	Referent			
≥12	17 (17.2 )	60 (30)	0.48			
Cigarette smoking						
Never	28 (28.3 )	166 (83)	Referent		Referent	
light smoker	19 (19.2 )	24 (12)	4.69 (2.3-9.7)	p <0.001	3.4 (1.3- 8.9)	0.014
heavy smoker	52 (52.5 )	10 (5)	30.82 (14.04-67.7)	p <0.001	15.8 (5.9-42.4)	p <0.001
Opium consumption						
Never	25 (25.2 )	171 (85.5)	Referent		Referent	
<18000 Gram - year in life time	19 (19.2 )	14 (7)	10.3 (4.8-22)	p <0.001	6 (2.3- 15.5)	p <0.001
≥18000 Gram - year in life time	55 (55.6)	15 (7.5)	26.3 (12.5-55.2)	p <0.001	11.3 (2.3-15.5)	p <0.001
Alcohol use				0.019	_	_
Never	90 (91.8 )	196 (98)	Referent			
Ever use	8 (8.2)	4 (2)	4.4 (1.3-14.8)			
Second hand exposure to smoking				0.02	_	_
No	59 (59.6)	146 (73)	Referent			
Yes	40 (40.4 )	54 (27)	1.8 (1.1-3)			
Second hand exposure to opium				0.22	_	_
No	71 (72.5)	156 (78.8 )	Referent			
Yes	27 (27.6)	42 (21.2 )	1.4 (0.8-2.5)			
History of work in industrial factories				0.04	_	_
No	86 (89.9)	192 (96)	Referent			
Yes	10 (10.1)	8 (4)	2.7 (1-7.1)			
Residing in proximity to industrial zone				0.02	_	_
< 2 km	81 (81.8 )	182 (91)	Referent			
≥ 2 km	18 (18.2 )	18 (9)	2.4 (1.1- 4.5)			
Exposure to chemical fertilizers				0.01	_	_
No	58 (58.6 )	145 (72.5)	Referent			
Yes	41 (41.4 )	55 (27.5)	1.9 (1.1- 3.1)			
Ever working as a driver				p < 0.001	_	_
No	76 (76.8 )	186 (93)	Referent			
Yes	23 (23.2 )	14 (7)	4 (2- 8.2)			
Ever working as a farmer				0.03	_	_
No	43 (43.4 )	113 (56.5)	Referent			
Yes	56 (56.6 )	87 (43.5)	1.7 (1-2.7)			
Ever working as a construction worker				0.001	_	_
No	77 (77.8 )	184 (92)	Referent			
Yes	22 (22.2 )	16 (8)	3.3 (1.6 -6.6)			
Use of grilled foods					_	_
< 1 time per week	58 (58.6 )	86 (43.4 )	Referent			
1-3 time per week	35 (35.4 )	102 (51.5)	0.5 (0.3-0.8)	0.009		
≥ 4 times per week	6 (6.6)	10 (5.1 )	0.9 (0.3- 2.6)	0.83		
Stuck-pot					_	_
< 1 time per week	61 (61.6 )	87 (43.9 )	Referent			
1-3 time per week	32 (32.3)	93 (47 %)	0.5 (0.3-0.8)	0.007		
≥4 times per week	6 (6.1 )	18 (9.1 )	0.5 (0.2- 1.3)	0.13		
carbonated drink and juices with preservatives					_	_
< 1 time per week	20 (20.2 )	63 (31.8 )	Referent			
1-3 time per week	32 (32.3 )	69 (34.9 )	1.5 (0.8- 2.8)	0.25		
≥4 times per week	47 (47.5 )	66 (33.3 )	2.2 (1.2- 4.2)	0.01		
Use of egg					_	_
< 1 time per week	16 (16.1 )	12 (6 %)	Referent			
1-3 time per week	60 (60.6 )	135 (67.8)	0.3 (0.1- 0.7)	0.008		
≥4 times per week	23 (23.2 )	52 (26.1 )	0.3 (0.1- 0.8)	0.016		
Cereals				0.11	_	_
1-3 time per week	7 (7.1 )	6 (3.1 )	Referent			
≥4 times per week	92 (92.9 )	193 (97 )	0.4 (0.1- 1.3)			
Pickle					_	_
< 1 time per week	64 (64.7 )	103 (52.1)	Referent			
1-3 time per week	28 (28.3 )	69 (34.9 )	0.7 (0.4- 1.1)	0.12		
≥4 times per week	7 (7.1 )	26 (13.1 )	0.4 (0.2- 1.1)	0.06		

## Discussion

We showed that cigarette smoking, opium consumption, and low level of income (<20 million Rials) independently increase the chance of bladder cancer. No relationship was found between diet, alcohol consumption, second-hand cigarette smoke, opium use, job, and the likelihood of bladder cancer.

We observed a strong dose-response relationship between the amount of opium consumption in gram-years and bladder cancer. The chance of bladder cancer increased 6 times among cases who used up to 18,000 gram-years in their lifetime. However, consumption of 18,000 gram-years opium and more increased the chance of bladder cancer by 11.3 times. The results of a systematic review and meta-analysis conducted in 2021 show a 3.8 times increase in chances of developing bladder cancer among opium users (Bidary et al., 2021), which is lower than our estimates. This may be due to differences in population selection, exposure definition and measurement, confounding factor control, and adjustments between individual studies included in this meta-analysis. Opium has been found to affect cancer through several mechanisms. A study conducted by Etemadi et al., (2020) showed that the concentration of polycyclic aromatic hydrocarbons (PAH), and volatile organic compounds (VOC) among exclusive opium users is higher than it is in non-users. Exposure to these chemicals has been associated with bladder cancer. Furthermore, Malaveille et al., (1982) demonstrated that pyrolysis of opium and its alkaloids may produce mutagens and possibly increase the risk of bladder cancer. Also, opium has displayed mutagenic activity in animal experiments, and sister chromatid exchanges have been observed in human peripheral blood lymphocytes (Perry et al., 1983). Increasing the methylation of DNA through the reduction of N-nitrosamines and N-nitrosodimethylamine through liver clearance was has been observed in association with morphine, one of the opium alkaloids (Hosseini et al., 2010; Karbakhsh et al., 2013). Urinary retention and consequently prolonged exposure of bladder with carcinogenic chemicals may increase the chance of bladder cancer, and opium impurities, such as lead and arsenic, may play a role in the carcinogenicity of opium (Schiff, 2002; Aghababaei et al., 2018). 

Similarly, we observed a dose-response pattern between the amount of lifetime cigarette smoking and bladder cancer. In our study, light smoking and heavy smoking were associated with a 3-time and a 15-time increased chance of bladder cancer, respectively. The higher risk of bladder cancer among cigarette smokers has been addressed in previous studies (Rink et al., 2015; Masaoka et al., 2016). At least 70 carcinogenic compounds such as aromatic amines and N-nitroso are known to exist in cigarettes. Cigarette-gene interactions involve DNA damage in the form of genomic events such as double-stranded breaks, base modifications, and bulky adduct formation (Stern et al., 2009; Jin et al., 2017). In line with previous studies (Lotfi et al., 2016; Weiner et al., 2018), we observed a reverse association between the level of income and bladder cancer, with higher income associated with a decreased likelihood of bladder cancer. High income may protect people against disease through greater access to medical services, better nutrition, and higher levels of awareness.

In contrast to some previous studies, we saw no association between occupation and bladder cancer. In a review conducted in 2020, the chances of developing bladder cancer were estimated to be 11.3 times greater than the general population; the study estimated that bus and heavy vehicle drivers, farmers, fishers, foresters, metalworkers, and welders were 6 times more likely to develop bladder cancer (Farmanfarma et al., 2020). Our bivariate analysis showed that working in industrial factories, residing in proximity to factories, exposure to chemical fertilizers, and working as drivers, as farmers, or as construction workers were associated with bladder cancer, but the findings didn’t last in multivariable-adjusted analysis. This finding suggests that the observed association between occupation and bladder cancer in previous studies may be explained by the confounding role of opium and cigarettes. The findings of an ecologic study on 160 countries demonstrated an association between opiate use and bladder cancer (Rashidian et al., 2016). Based on the national population size estimation of drug users in Iran in 2013, Kerman ranked fifth in terms of opium consumption and ranked third in terms of shire (combination of opium residue and pure opium) consumption (Nikfarjam et al., 2016). The high prevalence of opium use in Kerman province may explain the higher incidence of bladder cancer seen in the population-based cancer registry data (Shahesmaeili et al., 2018).

The present study’s findings provided important insights into the risk factors of bladder cancer, the most common cancer among men in Kerman province. In contrast to previous studies, we tried to measure all possible risk factors of bladder cancer and adjust the results to minimize any confounding effect based on a comprehensive literature review. Precise measurement of opium consumption and cigarette smoking is strength of the present study. However, as in all case-control studies, bias in the recall of past exposures may be an issue. 

The strong dose-response association between opium consumption, cigarette smoking, and bladder cancer highlights the need for extension of prevention and harm reduction programs, especially in regions with a high burden of disease. As opium users and cigarette smokers are at higher risk of bladder cancer, it is recommended that the cost-effectiveness of early screening and early detection policies.

## Author Contribution Statement

ASH and AB conceptualized the study. ASH, ZA, HP, MSH, HP designed the methodology and study instruments. ZA AND MM performed the data processing and analysis. ASH and ZA drafted the manuscript. All authors reviewed and edited the final manuscript and approved the submitted version.
